# Assessing the Safety of Sequential Radial Artery Grafting in Coronary Revascularization

**DOI:** 10.1016/j.atssr.2025.06.031

**Published:** 2025-07-30

**Authors:** AlleaBelle Bradshaw, Ifeanyi Chinedozi, Jace C. Bradshaw, Jonathan D. Mathews, Anson Y. Lee, Emily L. Larson, Hanghang Wang, Puja Kachroo, Zachary Darby, Jessica B. Briscoe, Jennifer S. Lawton

**Affiliations:** 1 Division of Cardiac Surgery, Department of Surgery, Johns Hopkins, Baltimore, Maryland; 2 Department of Anesthesiology and Critical Care, Johns Hopkins, Baltimore, Maryland; 3 School of Medicine, University of Hawaii, Honolulu, Hawaii; 4 Division of Cardiothoracic Surgery, Department of Surgery, Washington University in St. Louis, Saint Louis, Missouri

## Abstract

**Background:**

Use of the radial artery (RA) as a conduit during coronary artery bypass grafting is associated with better outcomes compared with vein. However, data on the RA as a sequential graft are limited. This study assessed the safety and efficiency of using sequential RA grafting.

**Methods:**

Patients with sequential vs nonsequential RA grafting by 1 surgeon from 2 hospitals from 2001 to 2022 were compared using propensity matching. Primary outcomes were total artery revascularization (TAR) and incomplete revascularization. Secondary outcomes included cardiopulmonary bypass and cross-clamp times, total number of arterial grafts, 30-day mortality, and complications. The Mann-Whitney *U* test, χ^2^ test, and propensity matching were used.

**Results:**

Of 517 patients who received RA grafting, 107 (20.7%) were sequential. After matching, there were 107 patients in the sequential group and 321 in the nonsequential group. Sequential RA use was associated with more TAR (*P* < .001) and less incomplete revascularization (*P* = .002). Matched patients with sequential RA grafting and 4 grafts had shorter bypass and cross-clamp times (*P* < .001). No differences were observed in clinical outcomes between matched groups.

**Conclusions:**

Patients with sequential RA grafting had more TAR with equivalent outcomes compared with those with single RA. These findings support the safety and efficiency of sequential RA grafting.

**In Short**

▪ Sequential radial grafting can increase total arterial grafting and decrease incomplete revascularization.
▪ Sequential radial grafting does not compromise outcomes.

Multiple arterial grafting is associated with excellent outcomes in patients undergoing coronary artery bypass grafting (CABG).^1^ Recent coronary revascularization guidelines recommended using the radial artery (RA) as the conduit of choice for the second most important coronary artery.^2^ This is supported by randomized trials demonstrating RA patency rates comparable to or better than the right internal mammary artery and vein.^3^

Despite recommendations for multiple arterial grafting,^2^ adoption has been poor in the United States (9.8%), with higher rates in Canada (10%-28%), Europe (3.9%-34%), and Asia (42%-52%).^4^ Cited barriers include concerns regarding wound complications and perceived longer operating time.^5^^,^^6^

Sequential RA grafting refers to the use of 1 RA graft with 1 proximal anastomosis but >1 distal anastomosis. RA patency and durability are not diminished with a sequential approach.^7^ This study evaluated the safety and efficiency of sequential RA grafting as a mechanism for increasing total arterial revascularization (TAR) without prolonging cardiopulmonary bypass (CPB) and cross-clamp times or compromising clinical outcomes. We hypothesize that sequential RA grafting is associated with increased TAR rates without worsening clinical outcomes.

## Patients and Methods

This study was approved by the Johns Hopkins and Washington University in St. Louis Institutional Research Boards. This is a retrospective cohort analysis of adults (≥18 years) undergoing first-time isolated CABG with at least 1 RA graft by a single surgeon. Data were collected from the 2 centers between 2001 and 2022. Patients with and without sequential RA grafting were compared. Patients with nonsequential RA grafts underwent grafting with 1 distal anastomosis, with the RA originating on the aorta, hood of a vein graft, or as a “T” from the left internal mammary artery. Patients with sequential RA grafting underwent grafting with 2 distal anastomoses with the RA originating on the aorta, hood of a vein graft, or as a “T” from the internal mammary artery. The first target anastomosis was performed side-to-side, and the second was end-to-side (Videos 1, 2).

Primary outcomes were TAR and incomplete revascularization (IR). TAR was defined by the total number of grafts received being equal to the number of arterial grafts. IR was defined as the difference between the total number of grafts and the number of diseased vessels. Secondary outcomes were the total number of arterial grafts, CPB and cross-clamp times, 30-day mortality, reintervention for myocardial infarction or bleeding, intensive care unit length of stay, 30-day readmission, and complications, which included stroke, renal failure, and pneumonia.

Propensity matching (1:3 ratio) was conducted, adjusting for potential confounders. Variables used for matching were chosen based on those identified as potential confounders using a directed acyclic graph: age, sex, body mass index, number of diseased vessels, and specific comorbidities (hypertension, peripheral vascular disease, diabetes, and prior myocardial infarction) (Supplemental Figure).^8^

Baseline characteristics and outcomes between groups were compared using Mann-Whitney *U* and χ^2^ tests. Continuous variables are reported as means with SDs. Categorical variables are reported as counts and percentages. *P* values of <.05 were considered significant.

## Results

Of 517 patients with RA grafting, 107 (20.7%) had sequential RA grafting. Unmatched patient characteristics are summarized in Table 1.

**Table 1:** Characteristics and Outcomes of Unmatched Patients With vs Without Sequential Radial Artery Grafting

Variables	All (N = 517)	Radial Artery Grafting		*P* Value
		Sequential (n = 107)	Nonsequential (n = 410)	
Characteristics				
Age, mean (SD), y	59.4 (8.3)	58.57 (8.9)	59.61 (8.1)	.248
Male sex	269 (71.4)	82 (75.7)	288 (70.2)	.321
BMI, mean (SD), kg/m^2^	30.8 (6.3)	29.0 (5.5)	31.2 (6.5)	**.001**
Diabetes	237 (45.8)	40 (37.4)	197 (48.0)	**.024**
Peripheral vascular disease	68 (13.2)	13 (12.1)	55 (13.4)	.721
Previous myocardial infarction	268 (51.8)	48 (44.9)	220 (53.7)	.108
Dyslipidemia	394 (91.6)	68 (89.5)	326 (92.1)	.604
Ejection fraction, mean (SD)	0.527 (0.12.6)	0.549 (0.119)	0.521 (0.128)	.041
Elective	389 (75.2)	69 (64.5)	320 (78.0)	.015
Diseased vessels				.110
0	2 (0.4)	0 (0.0)	2 (0.5)	
1	11 (2.1)	1 (0.9)	10 (2.4)	
2	104 (20.1)	14 (13.1)	90 (22.0)	
3	400 (77.4)	92 (86.0)	308 (75.1)	
Number of grafts	3.3 (0.9)	4.0 (0.6)	3.1 (0.8)	**<.001**
1	2 (0.4)	0 (0.0)	2 (0.5)	
2	96 (18.6)	0 (0.0)	96 (23.4)	
3	215 (41.6)	24 (22.4)	191 (46.6)	
4	167 (32.3)	64 (59.8)	103 (25.1)	
5	37 (7.2)	19 (17.8)	18 (4.4)	
Proximal				.561
Aorta	477 (92.3)	96 (89.7)	381 (92.9)	
Artery	11 (2.1)	4 (3.7)	7 (1.7)	
Vein	11 (2.1)	3 (2.8)	8 (2.0)	
CPB time, mean (SD), min	130.5 (38.4)	135.3 (31.0)	129.0 (40.4)	.146
Cross-clamp time, mean (SD), min	98.9 (29.8)	101.8 (26.3)	98.0 (30.8)	.268
Outcomes				
30-day mortality	7 (1.4)	1 (0.9)	6 (1.5)	1.000
30-day readmission	17 (8.6)	2 (2.9)	15 (11.6)	.061
Reoperation for				
Bleeding	10 (5.2)	3 (7.5)	7 (4.6)	.739
Myocardial infarction	2 (2.4)	1 (11.1)	1 (1.4)	.515
Pneumonia	4 (2.1)	0 (0.0)	4 (2.6)	.678
Renal failure	5 (2.6)	0 (0.0)	5 (3.3)	.546
Stroke	9 (4.7)	2 (5.0)	7 (0.7)	.872
ICU stay, mean (SD), h	40.7 (37.6)	35.8 (23.9)	42.0 (40.4)	.138
Ventilator time, mean (SD), h	6.2 (17.5)	4.0 (6.0)	6.9 (19.5)	.136
Any complications	192 (37.1)	40 (37.4)	154 (37.1)	.110

Unadjusted baseline characteristics and clinical outcomes of patients with vs without sequential radial artery grafting. Data are presented as n (%) unless indicated otherwise as mean (SD). Bold *P* values are statistically significant (*P* < .05).

BMI, body mass index; CPB, cardiopulmonary bypass; ICU, intensive care unit.

After matching, there were 428 total patients, and 107 (25%) had sequential RA grafting (Table 2). The group with sequential grafting had a lower percentage of elective cases and more total grafts compared with the nonsequential group. The aorta was the proximal origin in 399 (93%) of matched patients (Table 2). Sequential RA grafting was associated with increased TAR (52% vs 29%, *P* < .001) and decreased IR (0% vs 9%, *P* = .002) compared with nonsequential grafting (Table 3).

**Table 2:** Characteristics and Outcomes of Matched Patients With vs Without Sequential Radial Artery Grafting

Variables	All (N = 428)	Radial Artery Grafting		*P* Value
		Sequential (n = 107)	Nonsequential (n = 321)	
Characteristics				
Age, mean (SD), y	59.1 (8.4)	58.6 (8.9)	59.3 (8.2)	.471
Male sex	317 (74.1)	81 (75.7)	236 (73.5)	.750
BMI, mean (SD), kg/m^2^	29.4 (5.4)	29.0 (5.5)	29.5 (5.3)	.361
Diabetes	173 (40.4)	40 (37.4)	133 (41.4)	.178
Peripheral vascular disease	55 (12.9)	13 (12.1)	42 (13.1)	.934
Previous myocardial infarction	212 (49.5)	48 (44.9)	164 (51.1)	.267
Dyslipidemia	320 (91.7)	68 (89.5)	252 (92.3)	.578
Ejection fraction, mean (SD)	0.530 (12.6)	0.549 (0.119)	0.524 (0.127)	.077
Elective	317 (74.1)	69 (64.5)	248 (77.3)	**.031**
Diseased vessels				.628
0	0 (0.0)	0 (0.0)	0 (0.0)	
1	5 (1.2)	1 (0.9)	4 (1.2)	
2	68 (15.9)	14 (13.1)	54 (16.8)	
3	355 (82.9)	92 (86.0)	263 (81.9)	
Number of grafts	3.4 (0.85)	4.0 (0.6)	3.1 (0.8)	**<.001**
1	1 (0.2)	0 (0.0)	1 (0.3)	
2	67 (15.7)	0 (0.0)	67 (20.9)	
3	179 (41.8)	24 (22.4)	155 (48.3)	
4	145 (33.9)	64 (59.8)	81 (25.2)	
5	36 (8.4)	19 (17.8)	17 (5.3)	
Proximal				.331
Aorta	399 (93.2)	96 (89.7)	303 (94.4)	
Artery	9 (2.1)	4 (3.7)	5 (1.6)	
Vein	7 (1.6)	3 (2.8)	4 (1.2)	
CPB time, mean (SD), min	129.1 (36.6)	135.3 (31.0)	126.7 (38.3)	**.040**
Cross-clamp time, mean (SD), min	98.4 (29.2)	101.8 (26.3)	97.0 (30.2)	.170
Outcomes				
30-day mortality	3 (0.7)	1 (0.9)	2 (0.6)	1.000
30-day readmission	13 (7.6)	2 (2.9)	11 (10.8)	.111
Reoperation for				
Bleeding	8 (5.1)	3 (7.5)	5 (4.2)	.692
Myocardial infarction	2 (3.1)	1 (11.1)	1 (1.8)	.643
Pneumonia	4 (2.5)	0 (0.0)	4 (3.4)	.550
Renal failure	4 (2.5)	0 (0.0)	4 (3.4)	.550
Stroke	8 (5.1)	2 (5.0)	6 (5.1)	.843
ICU stay, mean (SD) h	40.3 (39.0)	35.8 (23.9)	41.8 (42.9)	.183
Ventilator time, mean (SD) h	6.0 (18.4)	4.0 (6.0)	6.7 (21.0)	.188
Any complications	158 (36.9)	40 (37.4)	118 (36.8)	1.000

Propensity-matched preoperative characteristics and postoperative clinical outcomes of patients with sequential vs nonsequential radial artery grafting. Data are presented as n (%) or as indicated otherwise as mean (SD). Bold *P* values are statistically significant (*P* < .05).

BMI, body mass index; CPB, cardiopulmonary bypass; ICU, intensive care unit.

**Table 3:** Grafting Strategies in Matched Patients With Sequential vs Nonsequential Radial Artery Grafting

Variable	Radial Artery Grafting		*P* Value
	Sequential (n = 107)	Nonsequential (n = 321)	
Total grafts, mean (SD), n	3.95 (0.64)	3.14 (0.82)	**<.001**
Arterial grafts, mean (SD), n	3.35 (0.50)	2.15 (0.39)	**<.001**
Total arterial revascularization	56 (52.3)	92 (28.7)	**<.001**
Incomplete revascularization	0 (0.0)	30 (9.3)	**.002**

Data presented as *n* (%) unless indicated otherwise as mean (SD). Bold *P* values are statistically significant (*P* < .05). Total arterial revascularization is defined as when total grafts received equal number of arterial grafts. Incomplete revascularization is defined as when total grafts received were less than the number of diseased vessels.

Matched patients with sequential grafting had longer CPB but not cross-clamp time vs nonsequential (Table 2). When matched patients were compared by number of grafts, patients who had 4 total grafts and sequential grafting had shorter CPB and cross-clamp times compared with nonsequential (135.6 vs 143.5 minutes, *P* = .014) and (101.3 vs 112.6 minutes, *P* < .001), respectively (Table 4). CPB and cross-clamp times were not significantly different among patients with other total number of grafts.

**Table 4:** Bypass and Cross-Clamp Times in Matched Patients With vs Without Sequential Radial Artery Grafting

Total grafts	Cardiopulmonary Bypass Time, min			Cross-Clamp Time, min		
	Sequential Radial (n = 107)	Nonsequential Radial (n = 321)	*P* Value	Sequential Radial (n = 107)	Nonsequential Radial (n = 321)	*P* Value
1	…	91		…	54	
2	…	94.7 (44.3)		…	72.5 (35.2)	
3	118.4 (32.2)	122.1 (29.3)	0.654	84.5 (28.0)	90.67 (24.08)	.329
4	135.6 (29.2)	143.5 (35.4)	0.0135	101.3 (24.1)	112.6 (24.8)	**<.001**
5	153.9 (25.5)	163.9 (28.6)	0.253	120.6 (18.6)	128.9 (19.9)	.169

Comparison of cardiopulmonary bypass and cross-clamp times in propensity-matched patients with vs without sequential radial artery grafting. Data are presented as mean (SD). Bold *P* values are statistically significant (*P* < .05).

## Comment

Whereas our previous work focused on the RA as a T graft from the internal mammary artery,^7^ most patients in this study had a RA graft originating from the aorta (93%), supporting the safety of this approach.

In this study, use of sequential RA grafting was associated with increased TAR and decreased IR. Given that TAR is linked to superior long-term outcomes^9^ and IR is associated with worse outcomes,^10^ these findings support the use of sequential RA grafting.

Sequential RA grafting did not increase cross-clamp time compared with nonsequential grafting, suggesting the technical complexity was similar. The increased CPB time in matched patients with sequential grafting is likely related to the increased total number of grafts (4 vs 3) rather than the time to perform sequential anastomoses. In fact, CPB and cross-clamp times were shorter with sequential grafting vs nonsequential in patients with 4 total grafts.

### Limitations

Limitations include the retrospective nature with inherent biases related to patient selection, procedures were performed by 1 surgeon, lack of long-term data and graft patency outcomes, and the RA sequential grafting subgroup was small.

### Conclusion

This study supports sequential RA grafting, offering a strategy to increase TAR, decrease IR, and improve efficiency without compromising outcomes.

## Figures and Tables

**Supplementary Figure 1:**
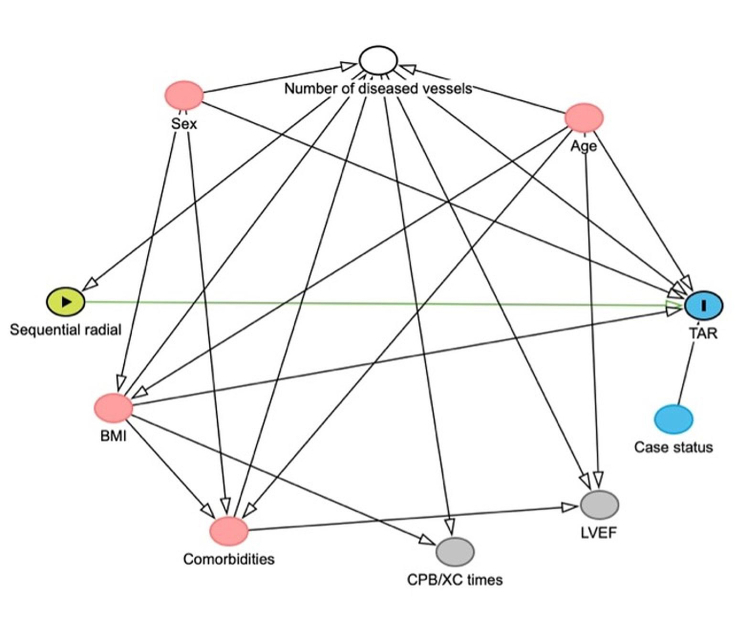


## References

[1] Sabik J.F., Mehaffey J.H., Badhwar V. (2024). Multiarterial vs single-arterial coronary surgery: 10-year follow-up of 1 million patients. Ann Thor Surg.

[2] Lawton J.S., Tamis-Holland J.E., Bangalore S. (2022). 2021 ACC/AHA/SCAI Guideline for Coronary Artery Revascularization: a report of the American College of Cardiology/American Heart Association Joint Committee on Clinical Practice Guidelines. J Am Coll Cardiol.

[3] Hamilton G.W., Raman J., Moten S. (2023). Radial artery vs. internal thoracic artery or saphenous vein grafts: 15-year results of the RAPCO trials. Eur Heart J.

[4] Gaudino M., Chikwe J., Falk V., Lawton J.S., Puskas J.D., Taggart D.P. (2020). Transatlantic editorial: the use of multiple arterial grafts for coronary revascularization in Europe and North America. Ann Thor Surg.

[5] Velez A.K., Alejo D., Holmes S.D. (2023). Multiple arterial graft use in coronary artery bypass surgery: surgeon perspective vs practice. Ann Thor Surg.

[6] Velez A.K., Canner J.K., Etchill E. (2021). Measures to increase use of multiple arterial grafts for isolated coronary artery bypass grafting. J Am Coll Surg.

[7] Barner H.B., Bailey M., Guthrie T.J. (2012). Radial artery free and T graft patency as coronary artery bypass conduit over a 15-year period. Circulation.

[8] Gongola A., Bradshaw J.C. (2023). Directed acyclic graphs in surgical research. J Surg Res.

[9] Gaudino M., Bakaeen F.G., Benedetto U. (2019). Arterial grafts for coronary bypass: a critical review after the publication of ART and RADIAL. Circulation.

[10] Gaba P., Gersh B.J., Ali Z.A., Moses J.W., Stone G.W. (2021). Complete versus incomplete coronary revascularization: definitions, assessment and outcomes. Nat Rev Cardiol.

