# Supplementary material for: Assessing the Safety of Sequential Radial Artery Grafting in Coronary Revascularization
Source: Ann Thorac Surg Short Rep. 2025 Jul 30;3(4):1000–4. doi: 10.1016/j.atssr.2025.06.031 (PMC12712208; doi:10.1016/j.atssr.2025.06.031)
Supplement: Supplementary Material [file mmc1.docx]

**Supplemental Figure 1:** Directed acyclic graph demonstrating relationships between variables

**Legend**: Directed acyclic graphs demonstrate relationships between variables.^8^ Sex, number of diseased vessels, age, and comorbidities (hypertension, peripheral vascular disease, diabetes, and prior myocardial infarction) were identified as potential confounders and were therefore used for propensity matching. Ejection fraction, cardiopulmonary bypass time, and cross-clamp time were identified as mediators and were not used for propensity matching. Figure created using daggity.net.

BMI, body mass index; CPB, cardiopulmonary bypass; LVEF, left ventricular ejection fraction; RA, radial artery; XC, cross-clamp

**Supplemental Videos**

**Video 1**: Cardiac catheterization after sequential radial artery grafting

**Video 2**: Intraoperative video demonstrating sequential radial artery grafting
